# Secondary prevention of osteoporosis in non-neck of femur fragility fractures: is it value for money? A retrospective, prospective and cross-sectional cohort study

**DOI:** 10.1186/1749-799X-8-44

**Published:** 2013-12-01

**Authors:** Tamer Mettyas, Clare Carpenter

**Affiliations:** 1 Orthopaedic Department, Royal Brisbane and Women's Hospital, Brisbane, Australia; 2 Orthopaedic Department, University Hospital of Wales, Heath Park, Cardiff CF14 4XW, UK

**Keywords:** Fragility fractures, Bisphosphonates, Osteoporosis, Non-NOF fractures, DEXA, NICE guidelines

## Abstract

**Background:**

Osteoporosis is one of the commonest bone diseases in which bone fragility is increased. Over 300,000 patients present to hospitals in the UK with fragility fractures each year, with medical and social care costs - most of which relate to hip fracture care - at around £2 billion. The number of these fractures rises by 2% a year. The 30 days mortality is 10% and 30% at 1 year. The purpose of this study is to review the current practice according to NICE and BOA guidelines of secondary prevention of osteoporosis and to suggest changes to these guidelines.

**Methods:**

Patients over 50 years old admitted as inpatients to our facility with non-neck-of-femur (NOF) fragility fractures in March and September 2008 were studied. Retrospectively (March), looking for risk factors and if treated or not, then prospectively (September), after introducing the new trauma admission sheet. Also cross-sectional study was performed by comparing the services provided for NOF and non-NOF fragility fractures in September. Two-sample *t* test is used to compare between percentages.

**Results:**

Twenty-nine percent of fragility fractures are non-NOF fractures with a mean age of 70 years, while the remaining 71% are NOF fractures with a mean age of 80 years. There is a great difference in the care provided to these patients: non-NOF fragility fractures got less attention for assessment of osteoporosis (25%) and obtained less interest in investigations by medical staff (11%) and, finally, less intentions to treat osteoporosis (35%), compared to NOF fractures in which 35% of cases were assessed, 47% were investigated and 71% were treated for osteoporosis. Twenty-five percent of NOF fracture patients were found to have previous fragility fractures in the preceding years, while only 6% were on osteoporosis treatment before the fracture.

**Conclusion:**

Osteoporosis (a new epidemic) is the most common disease of the bone and its incidence is rising rapidly as the population ages. Though treatable, it is often left untreated. We believe that treating patients with *non*-*NOF fragility fractures* from osteoporosis *before* proceeding to NOF fractures would improve their quality of life and reduce the burden on hospital services and funding.

## Background

Osteoporosis is the most common disease of bone (new epidemic) [1]. It is a state in which the bone is fully mineralized but its structure is abnormally porous and its strength is less than normal for a person of that age and sex, and this is accompanied by increased fragility [2]. It is estimated that 1.2 million women in the United Kingdom have osteoporosis (*T*-score <2.5) [3]. Over 300,000 patients present to hospitals in the UK with fragility fractures each year, with medical and social care costs - most of which relate to hip fracture care - at around £2 billion [1]. In 2007, 70,000 people over 60 years of age suffered a hip fracture; the number rises by 2% a year. Current projections suggest that, in the UK, hip fracture incidence will rise to 91,500 in 2015 and 101,000 in 2020. Currently, the annual UK healthcare costs alone (at £7000–£12 000 per patient fracture), are £1.8 billion. The 30 days mortality is 10% and 30% at 1 year [1]. In Australia, currently 2.2 million Australians have osteoporosis and this is expected to increase to 3 million by 2021 [4,5].

Despite the known impact of fragility fractures, osteoporosis still remains unrecognised and untreated in over 50% of patients who present with fragility fractures. Orthopaedic surgeons are often the first to encounter these patients who present with fragility fractures [6].

### UK standards

National Institute for Health and Clinical Excellence (NICE) published the guidelines and recommendations (issued 2008, last update 2011): use of bisphosphonates, selective oestrogen receptor modulators (raloxifene) and/or parathyroid hormone (teriparatide) to reduce the risk of further osteoporosis-related fractures in women who have gone through the menopause and who have already had an osteoporosis-related fracture [3]. Treatment is offered to prevent further fractures that will depend on the patient's age, her bone density and how many risk factors for fracture she has [3]. Alendronate is recommended as a possible treatment to prevent bone loss in postmenopausal women who have already had a fracture and have had osteoporosis diagnosed. If a woman cannot take alendronate, risedronate and etidronate are recommended under certain circumstances as possible alternative treatments to prevent further fractures. If a woman cannot take alendronate or either risedronate or etidronate, then strontium ranelate and raloxifene are recommended under certain circumstances as possible alternative treatments to prevent further fractures. If a woman cannot take alendronate or either risedronate or etidronate, or strontium ranelate, teriparatide is recommended under certain circumstances as a possible alternative treatment to prevent further fractures. Teriparatide is also recommended as a possible alternative treatment for a woman who has another fracture when she has been taking alendronate, risedronate or etidronate for 1 year and her bone density has fallen [3]. The guidance says that women who are 75 or over may not need a bone scan to diagnose their osteoporosis [3]. Sustaining a fragility fracture at least doubles the risk of future fractures; that risk remains for 10 years and is greatest in the first year after the original fragility fracture [7].

The British Orthopaedic Association (BOA) published the blue book (September 2003, updated 2007) - *The care of patients with fragility fractures* - and suggests the following components to prevention [1]: (a) assessment and treatment of osteoporosis, (b) prevention of falls, (c) prevention of hip fractures by use of hip protectors, and (d) ‘A Fracture Liaison Service’, delivered by a nurse specialist, which is a proven approach.

The purpose of this study is to assess the current practice according to NICE and BOA guidelines of the secondary prevention of osteoporosis and to suggest changes to these guidelines.

## Methods

Our research is a cohort study conducted in Morriston Hospital in Swansea (from May to October 2008), updated with literature review in 2013. Ethical approval was not required for this study.

All patients over 50 years old admitted (in our institution: Morriston Hospital, Swansea, UK) with non-neck-of-femur fracture, in March and September 2008, were identified and studied. Retrospectively, March admissions were checked for risk factors and noted if appropriately treated or not. Prospectively, after the introduction of a screening *pro forma*, September admissions were studied in the same way. Cross-sectional study is used to compare the neck-of-femur (NOF) to non-NOF fragility fractures (in September).

This was done by checking the following: (1) patients' score (for osteoporosis risk), (2) appropriate treatment initiated in the hospital and (3) patients who continued treatment after discharge.

The following are the inclusion criteria:

1. Patients presenting with non-neck-of-femur fragility fractures

2. Over 50 years of age

3. Admitted to hospital as inpatient in March and September 2008

Out of 89 patients over 50 years old, with fractures admitted from Accident and Emergency (A & E) in September 2008, *83* files of patients were studied. Fifty patients were admitted with NOF fractures (60%), in which all proximal femoral fractures are included in this category, while 33 patients had non-NOF fragility fractures (40%).

In March 2008, there were 60 patients admitted with fractures mostly from A & E and few from fracture clinic with non-NOF fragility fractures. Randomly, 37 cases were studied, and 5 of these were excluded due to incomplete information.

We aimed to assess the current practice with regards to the guidelines of secondary prevention of osteoporosis for hospital admissions. The statistical test used for comparison is the two-sample *t* test (between percents) and the *p* value calculated is a two-tailed probability.

## Results

The results are presented in Tables 1,2,3,4,5,6,7,8,9,10,11.

**Table 1:** Total admissions studied (within our criteria)

	**March (non-NOF fragility fractures)**	**September (non-NOF fragility fractures)**	**September (NOF fractures)**
Number of patients admitted	32	33/83 (40%)	50/83 (60%)

**Table 2:** Average age (years)

	**March (non-NOF fragility fractures)**	**September (non-NOF fragility fractures)**	**September (NOF fractures)**
Average age	70.7 (50–89)	69 (50–101)	80 (56–97)

Values in parentheses refer to range.

**Table 3:** Male/female ratio and percentage of female to total admissions (over 50 years old with fractures)

	**March (non-NOF fractures)**	**September (non-NOF fractures)**	**September (NOF fractures)**
Male/female	10/22	15/18	19/31
Percentage of female to total admissions	68.7	54.5	62

**Table 4:** Percentages of fragility fractures to all fractures and in each sex

		**March (non-NOF fragility fractures)**	**September (non-NOF fragility fractures)**	**September (NOF fractures)**
Fragility fractures/total fractures admitted		23/32 (72)	20/33 (61)	48/50 (96)
Fragility fractures/total fractures admitted (breakdown by gender)	Male	5/10 (50)	7/15 (47)	17/19 (89)
	Female	18/22 (82)	13/18 (72)	31/31 (100)

Values are presented as number/total (percentage).

**Table 5:** Site of fracture (non-NOF fragility fractures)

	**March**	**September**	**Total**
Upper limb	15/32 (47)	14/33 (42)	29/65 (44.6)
Lower limb (non-NOF, non-pelvis)	12/32 (37.5)	18/33 (55)	30/65 (46.2)
Pelvis	3/32 (9)	1/33 (3)	4/65 (6.2)
Vertebrae	2/32 (6.5)	0/33 (0)	2/65 (3)

Values are presented as number/total (percentage).

**Table 6:** Percentage of patients having previous fragility fractures

	**March (non-NOF fragility fractures)**	**September (non-NOF fragility fractures)**	**September (NOF fractures)**
Patients with previous fragility fractures	Not assessed	7/20 (35)	12/48 (25)

Values are presented as number/total (percentage).

**Table 7:** Osteoporosis assessment (compliance by staff) in September (via our new purple admission sheet)

	**September (for all fractures NOF and non-NOF)**
Patients assessed for osteoporosis	22/83 (26.5)

Values are presented as number/total (percentage).

**Table 8:** Percentage of DEXA requested for patients <75 years with fragility fractures

	**March (non-NOF fragility fractures)**	**September (non-NOF fragility fractures)**	**September (NOF fractures)**
DEXA requested for admitted patients under 75 with fragility fractures	2/12 (16.5)	1/9 (11)	7/15 (47)

Values are presented as number/total (percentage). DEXA, dual*-*energy X-ray absorptiometry.

**Table 9:** Commencement on treatment for osteoporosis in patients with fragility fractures

	**March (non-NOF fragility fractures)**	**September (non-NOF fragility fractures)**	**September (NOF fractures)**
Already on treatment	1	3	3 (6%)
On admission	1	4	7
Started later in hospital	3 (average starting is 4 days after admission)	0	24 (average starting is 2.7 days after admission)
No treatment (treatment missed)	18	13	14 (probably included in the study before orthogeriatrics commence treatment)
Total	23/23	20/20	48/48
Treated patients (*n* (%))	5/23 (22)	7/20 (35)	34/48 (71)

**Table 10:** Type of treatment

	**March (non-NOF fragility fractures)**	**September (non-NOF fragility fractures)**	**September (NOF fractures)**
Calcium +/− vit D only	2	3	11
Bisphosphonate, strontium or teriparatide	1	0	1
Both	2	4	22
Total	5	7	34

**Table 11:** Comparison between non-NOF fragility fractures and NOF fractures in September 2008

	**Non-NOF fragility fractures**	**NOF fractures**
Mean age	70	80
Male/female ratio having fragility fracture	12:31 (39:100)	17:31 (55:100)
Cases (%)	40	60
Fragility fractures (*n* (%))	20/68 (29)	48/68 (71)
Patients assessed for osteoporosis (by completion of the new admission sheet) (*n* (%))	5/20 (25)	17/48 (35)
Requested DEXA (%)	11	47
Treated patients (*n* (%))	7/20 (35)	34/48 (71)

While the majority of non-NOF fractures are females (65%), about one third of fragility fractures over 50 years old are males (Tables 3,4,11).

Twenty-five percent of patients with NOF fragility fractures had prior non-NOF fragility fractures. Only 6% of the NOF fragility fracture patients were on osteoporosis treatment before the new fracture (Tables 6 and 9).

The introduction of the new trauma admission sheet in our institution in August 2008 showed some improvement in the care provided for non-NOF fragility fractures (Table 9).

There is a great difference in the care provided to the patients with fragility fractures between non-NOF and NOF fractures. Unfortunately, non-NOF fractures got less attention for assessment of osteoporosis (25%) compared to 35% for NOF, less interest in investigations (11%) compared to 47% for NOF and finally, less intention to treat (35%) compared to 71% for NOF (Table 11).

## Discussion

Based on the above results, the following points are raised:

•NICE guidelines did not include about one third of the cases which comprises the male ratio in fragility fractures over 50 years old. No recommendation was made for the length of osteoporosis treatment.

•Morbidity, mortality and loss of independence after major fragility fracture are greater in men than women [8].

•The introduction of the new trauma admission sheet in our institution in August 2008 showed some improvement in the care provided for non-NOF fragility fractures. As in September, there was a remarkable improvement regarding history taking, proper description of the mechanism of injury, previous fragility fractures and osteoporosis assessment. On the other hand, there was no significant difference in the treatment provided for osteoporosis of these fragility fractures (22% in March and 35% in September).

•There is a great difference in the care provided for patients with fragility fractures between non-NOF and NOF fractures. Unfortunately, non-NOF fractures have got less attention for assessment of osteoporosis (i.e. 25% - compared to 35% for NOF - *p* value 0.42), less interest in investigations (i.e. 11% - compared to 47% for NOF - *p* value 0.006) and finally less intentions to treat (i.e. 35% - compared to 71% for NOF - *p* value 0.007) (the statistical test used for comparison is: 2 sample *t* test between percents). The *p* value calculated is two-tailed probability (Table 11).

•While 25% of NOF fragility fracture patients had previous non-NOF fragility fractures, only 6% of the NOF fractures patients were on osteoporosis treatment (Table 9).

•Non-NOF fractures are about 29% (of the total number of fragility fractures studied in our inclusion criteria) with a mean age of 70 years, compared to NOF fractures, which are 71% with a mean age of 80 years. The majority of non-NOF fractures are females (65%) (Table 11).

•NICE guidelines did not include the non-NOF fragility fracture patients as crucial targets for osteoporosis assessment and treatment.

•This research is limited to the inpatient admissions as better documentation and care would be expected. On the other hand, the vast majority of patients with non-neck-of-femur fragility fractures are seen in fracture clinics and to a less extent in GP practice.

•In our study we found that the osteoporosis targets are clearly not being reached. Risk stratification is not being undertaken and cost-effective secondary prevention is not being utilised effectively. Below target results were also found in other studies and researches [9].

### Recommendations

Based on the above findings, we would like to suggest the following recommendations:

1. Improve osteoporosis assessment and treatment by

(a) Education for new juniors at induction

(a) Highlighting for seniors

(a) Introduction of clinical pathway including assessment sheet would be beneficial if well designed

(a) Inclusion of men in the guidelines would be of significance

(a) Considering remuneration for the institute, hospital or GP practice for recruiting suitable patients and commencing osteoporosis treatment

(a) Continuous practice monitoring and regular auditing

2. New recommendations:

(a) Inclusion of men in the guidelines of assessment and treatment of osteoporosis

(a) Treating *non-NOF fragility fractures* from osteoporosis *before* proceeding to NOF fractures

3. Considering the recruitment of nurse specialists into fracture clinics for A Fracture Liaison Service, which is a proven approach to the identification, assessment and treatment of fracture risk [1,10]

4. Develop liaisons with interested general practitioners, the nearest osteoporosis lead physician and falls service

5. Consider arranging sessions for these patients in fracture clinics for advice and initiation of osteoporosis treatment (by the specialist nurse) [1]

6. Coordination and teamwork between orthopaedics and orthogeriatrics

## Conclusions

Osteoporosis (a new epidemic) is the most common disease of the bone and its incidence is rising rapidly as the population ages. Though treatable, it is often left untreated. Non-NOF fragility fractures got less attention for investigation and treatment of osteoporosis compared to NOF fragility fractures.

The guidelines for assessment and treatment of osteoporosis should include non-NOF fragility fractures as crucial targets before they proceed to NOF fractures. These measures would improve the quality of life for patients and would reduce the medical and social care costs by decreasing the number of NOF fragility fracture cases.

Men represent about one third of fragility fracture patients over 50 years old. Also, Morbidity, mortality and loss of independence after major fragility fracture are greater in men than women. We recommend the inclusion of men in the guidelines for assessment and treatment of osteoporosis.

Better coordinated services (offering early diagnosis and bone protection, optimal fracture care and secondary prevention) would improve quality of life for patients and reduce the burden on hospital services and government funding *especially by treating non-NOF fragility fractures from osteoporosis before proceeding to NOF fractures.*

## Abbreviations

A & E: Accident and emergency; BOA: British Orthopaedic Association; DEXA: Dual*-*energy X-ray absorptiometry; GP: General practitioner; NICE: National Institute for Health and Clinical Excellence; NOF: Neck of femur; UK: United Kingdom; Vit D: Vitamin D; %: Percentage.

## Competing interest

The authors declare that they have no competing interests.

## Authors’ contributions

TM collected and analysed the data and wrote the manuscript. CC supervised the research and reviewed the manuscript. Both authors read and approved the final manuscript.
